# Highly interconnected genes in disease-specific networks are enriched for disease-associated polymorphisms

**DOI:** 10.1186/gb-2012-13-6-r46

**Published:** 2012-06-15

**Authors:** Fredrik Barrenäs, Sreenivas Chavali, Alexessander Couto Alves, Lachlan Coin, Marjo-Riitta Jarvelin, Rebecka Jörnsten, Michael A Langston, Adaikalavan Ramasamy, Gary Rogers, Hui Wang, Mikael Benson

**Affiliations:** 1 The Centre for Individualized Medication, Linköping University Hospital, Linköping University, Linköping, SE-58185, Sweden; 2 MRC-Laboratory of Molecular Biology, University of Cambridge, Hills Road, Cambridge, CB2 0QH, UK; 3 Department of Genomics of Common Disease, School of Public Health, Imperial College, London, W2 1PG, UK; 4 Department of Child and Adolescent Health, National Institute of Health and Welfare, University of Oulu, Oulu, FI-90101, Finland; 5 Mathematical Sciences, Chalmers University of Technology, University of Gothenburg, Gothenburg, SE-412 96, Sweden; 6 Department of Electrical Engineering and Computer Science, University of Tennessee, Knoxville, TN 37996-2250, USA; 7 Respiratory Epidemiology and Public Health Group, National Heart and Lung Institute, Imperial College, London, SW7 2AZ, UK; 8 Unit for Paediatric Allergology, Queen Silvia Children's Hospital, Gothenburg, SE-416 85 Sweden

## Abstract

**Background:**

Complex diseases are associated with altered interactions between thousands of genes. We developed a novel method to identify and prioritize disease genes, which was generally applicable to complex diseases.

**Results:**

We identified modules of highly interconnected genes in disease-specific networks derived from integrating gene-expression and protein interaction data. We examined if those modules were enriched for disease-associated SNPs, and could be used to find novel genes for functional studies. First, we analyzed publicly available gene expression microarray and genome-wide association study (GWAS) data from 13, highly diverse, complex diseases. In each disease, highly interconnected genes formed modules, which were significantly enriched for genes harboring disease-associated SNPs. To test if such modules could be used to find novel genes for functional studies, we repeated the analyses using our own gene expression microarray and GWAS data from seasonal allergic rhinitis. We identified a novel gene, *FGF2*, whose relevance was supported by functional studies using combined small interfering RNA-mediated knock-down and gene expression microarrays. The modules in the 13 complex diseases analyzed here tended to overlap and were enriched for pathways related to oncological, metabolic and inflammatory diseases. This suggested that this union of the modules would be associated with a general increase in susceptibility for complex diseases. Indeed, we found that this union was enriched with GWAS genes for 145 other complex diseases.

**Conclusions:**

Modules of highly interconnected complex disease genes were enriched for disease-associated SNPs, and could be used to find novel genes for functional studies.

## Background

Medical research often focuses on individual diseases and genes. However, complex diseases show considerable comorbidity and are associated with altered interactions between thousands of genes. This suggests a need to find generally applicable principles to study multiple diseases and genes. One solution may be to map differentially expressed, disease-associated genes on to the human protein-protein interaction (PPI) network. Gene expression microarray studies of several complex diseases have shown that differentially expressed genes tend to form modules of interacting and functionally related genes [1-5]. Those modules may help to identify genes harboring disease-associated SNPs [6]. The identification is, however, complicated by the involvement of multiple modules in the same complex disease.

In this study, we hypothesized that modules containing the most interconnected complex disease-associated genes would be enriched for disease-associated SNPs (note that highly interconnected disease genes have many interactions with other disease genes, while hub genes have interactions with any other gene). This hypothesis was based on recent discoveries in network medicine. Firstly, the effects of disease-associated SNPs tend to propagate through the PPI network, affecting the local neighborhood around the SNP-harboring genes [7-9]. Secondly, genes harboring disease-associated SNPs tend to form modules in the PPI network [10-13]. Those studies are mainly based on rare hereditary diseases. A recent meta-analysis of genome-wide association studies (GWAS), however, suggests that genes harboring disease-associated SNPs in complex diseases are also highly interconnected [14]. Taken together, previous studies showed that genes harboring disease-associated SNPs tend to form modules, and that the same is true for differentially expressed genes [15]. In this study, we integrated these two findings and used modules formed by differentially expressed disease genes to find genes harboring disease-associated SNPs. For this, first we defined modules in disease-specific networks for 13 complex diseases and show that these highly interconnected genes in these modules are enriched for disease genes identified through GWAS. To test the general applicability of our findings, the selected diseases were highly diverse and included oncological, metabolic and inflammatory diseases. Using in-house generated gene-expression and GWAS data, we showed that such modules could be used for identifying novel genes for functional studies, using seasonal allergic rhinitis (SAR) as a disease model. Finally, we show that overlapping modules of the complex diseases are generally enriched for genes harboring disease-associated SNPs, especially pleiotropic genes, identified by GWAS of 145 complex diseases.

## Results

### Disease-specific core susceptibility modules are enriched for disease-associated genes in 13 complex diseases

**Figure 1:**
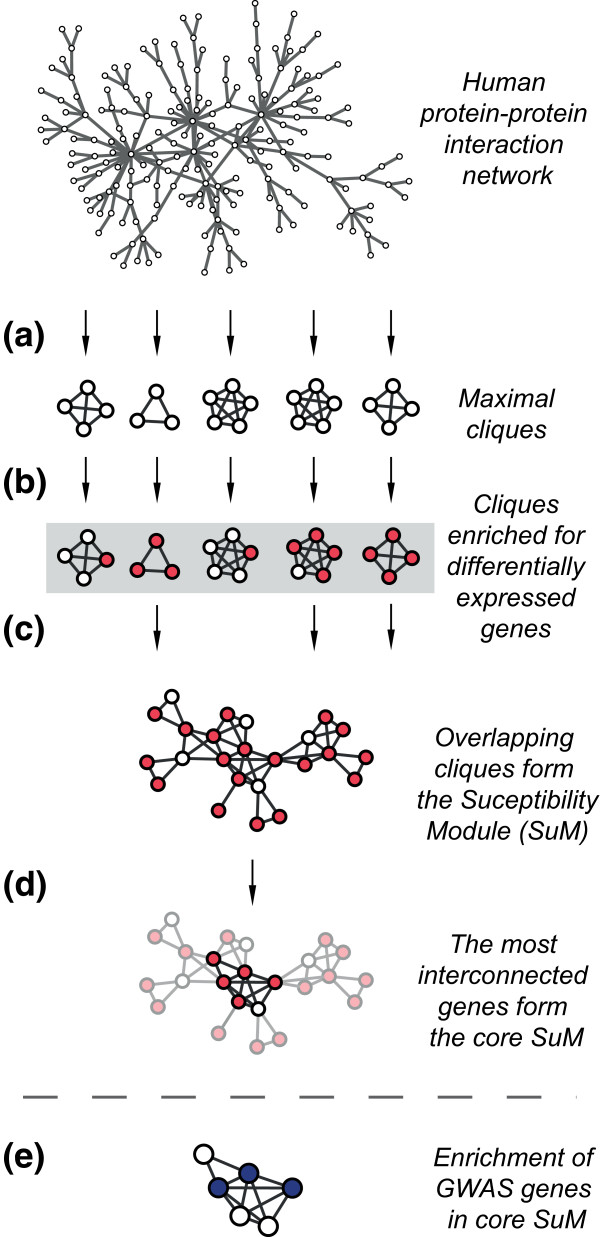
**Overview of identification of susceptibility modules (SuMs)**. **(a) **Maximal cliques were obtained from a human PPI. **(b) **Disease-associated cliques were identified by selecting those that were enriched for differentially expressed genes. **(c) **Such cliques were mapped onto the PPI network, resulting in the identification of a SuM of overlapping cliques. **(d) **A core SuM was identified using average shortest path length. **(e) **This core SuM was validated by showing enrichment for GWAS genes.

**Figure 2:**
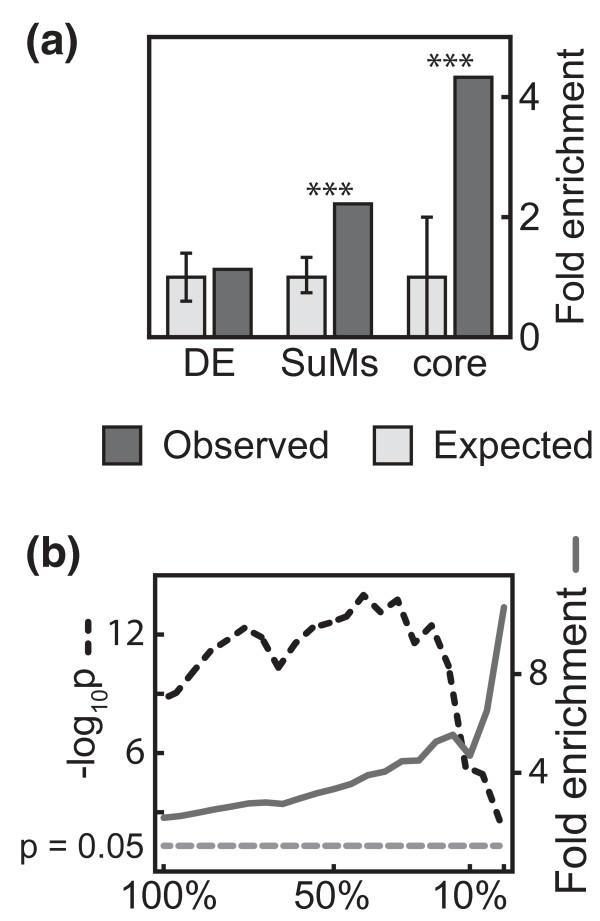
**Enrichment of GWAS genes from 13 oncological, immunological and metabolic diseases in SuMs and core SuMs**. **(a) **The enrichment of GWAS genes in core SuMs was stronger when the cutoff was more restrictive. The 10% cutoff was chosen to define core SuMs. **(b) **SuMs and core SuMs show strong enrichment of GWAS genes compared to differentially expressed (DE) genes. Error bars represent the 95% confidence interval of the randomized selections. Asterisks represent p < 0.001.

We defined modules in disease-specific networks by using (a) the global human PPI network and (b) differentially expressed genes for each disease. Those modules will henceforth be referred to as susceptibility modules (SuMs). Genes with high interconnectivity in the SuMs were defined as core SuMs (Figure 1; see Materials and methods). We examined whether the disease-specific core SuMs were enriched for genes harboring disease-associated SNPs by analyzing complex diseases for which gene expression microarray data from relevant cells or tissues were available in the public domain, and where GWAS had identified genes harboring disease-associated SNPs. Such genes will henceforth be referred to as GWAS genes. Thirteen oncological, immunological or metabolic diseases fulfilled these criteria (Table 1; Additional file 1). For each disease, we derived a SuM and within it, a core SuM. The enrichment of GWAS genes in the core SuMs was 4.71-fold compared to the whole PPI network (*P *< 10^-5^). The corresponding figure of the SuMs was 2.22-fold (*P *< 10^-5^). In contrast, using only differentially expressed genes we found a mere 1.15-fold enrichment of GWAS genes (*P *= 0.3; Figure 2a). We tested different cutoffs for interconnectivity. We found that increasingly stringent cutoffs for core SuMs were associated with stronger enrichment of GWAS genes (Figure 2b). Based on these analyses we defined core SuMs as the 10% of the SuM genes with the lowest average shortest path length. This demonstrated the effectiveness of modules to identify GWAS genes, compared to differentially expressed genes.

**Table 1:** Overview of SuMs in 13 complex diseases

			GWAS genes in PPI	GWAS genes in SuM		GWAS genes in core SuM	
					
Disease	GEO accession	Genes in SuM	network	Observed	Expected	Observed	Expected
Asthma	GSE4302	587	8	1	0.39	0	0.04
Breast cancer	GSE10810	2,474	7	2	1.45	1	0.15
Chronic lymphocytic leukemia	GSE8835	1,787	11	3	1.64	0	0.17
Colorectal cancer	GSE9348	1,651	5	3	0.69	1	0.07
Crohn's disease	GSE6731	1,458	16	5	1.95	3	0.20
Lung adenocarcinoma	GSE7670	2,524	10	4	2.11	0	0.21
Obesity	GSE12050	2,268	13	4	2.47	0	0.25
Parkinson's disease	GSE20141	1,871	21	5	3.29	1	0.33
Prostate cancer	GSE6919	751	16	3	1.01	1	0.10
Psoriasis	GSE13355	2,274	12	8	2.28	1	0.23
Schizophrenia	GSE17612	1,586	18	4	2.39	0	0.24
Type 2 diabetes	GSE20966	1,658	21	5	2.91	2	0.29
Ulcerative colitis	GSE6731	2,365	25	13	4.95	3	0.50
Total	-	-	-	60	27.53	13	2.76

Gene expression dataset for each disease obtained from the NCBI Gene Expression Omnibus (GEO).

### Core SuMs could be used to find novel genes for functional studies

We then determined whether core SuMs could be used to find novel genes for functional studies. We repeated the analyses using our own gene expression microarray and GWAS data from patients with SAR. This is an ideal model of complex diseases because it is possible to mimic and analyze the disease process in allergen-challenged cells from patients [16] (Extended background in Additional file 2). Novel genes can be functionally examined by combining small interfering RNA (siRNA) and gene expression microarrays in Th2 polarized cells [17-19], (Extended background in Additional file 2).

We constructed a SuM and a core SuM for SAR using differentially expressed genes obtained by performing gene expression microarray analysis of allergen-challenged CD4+ cells in samples from 12 patients as previously described [19,20] (Additional file 3). The SuM included 622 of the 2,822 differentially expressed genes in the PPI network, and 1,191 genes in total (Figure 1a in Additional file 4). Next, we tested if they could be replicated in the additional study material. The repeated analyses resulted in highly similar SuMs and core SuMs (*P *< 10^-15 ^in both cases, determined by a χ^2^-test; Figure 1b in Additional file 4). We also compared gene expression microarray data from allergen-challenged CD4+ cells from patients with SAR and allergen-challenged CD4+ cells from healthy controls and found differences in disease-relevant pathways and genes (Extended results in Additional file 2).

To determine whether the genes in the SuM and core SuM were enriched for SAR-associated SNPs, we analyzed an independent GWAS of 4,772 individuals in the North Finland Birth Cohort (Figure 1e). We found that intragenic SNPs within the 119 genes in the core SuM were 3.4 times more likely to be disease-associated than expected by chance (*P *= 1 × 10^-5^). This led to the identification of two novel genes, *FGF2 *and *MAPK8 *(Additional file 5). These findings were appropriately supported by false discovery rate calculations (Extended results in Additional file 2; Additional file 6). These genes had not been previously associated with SAR [21]. While *MAPK8 *has a known role in type 1 allergic inflammation [22-24], *FGF2 *is a novel gene. We tested the functional relevance of *FGF2 *by siRNA-mediated knock-down of this gene in Th2 polarized cells, followed by gene expression microarrays (Extended methods and Extended results in Additional file 2; Additional file 7). The knock-down resulted in altered expression of several pathways of potential relevance for type 1 allergic inflammation, as well as individual genes of known relevance for type 1 allergic inflammation, including *MAFB *and *NFKB1 *[25-30] (Additional file 8). In contrast to the core SuM, no enrichment of SNPs was found in the SuM.

### The union of core SuMs of different diseases was enriched for pleiotropic genes

**Figure 3:**
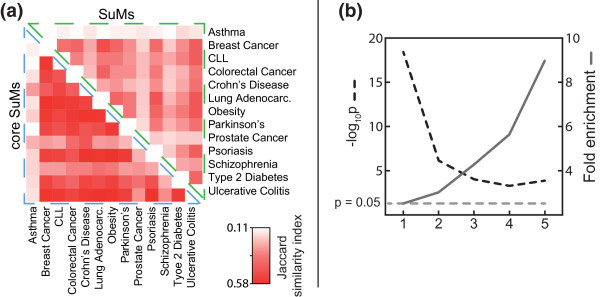
**Similarity of core SuMs of different diseases and enrichment of disease genes in the union of core SuMs**. **(a) **Heatmap showing that core SuMs are more similar than SuMs. The color intensity represents similarity, defined by computing the ratio between the number of genes shared by two SuMs and the total number of genes in the two SuMs (Jaccard similarity index). **(b) **In an extended analysis of GWAS genes of 145 other diseases, the union of the core SuMs was enriched with GWAS genes. This enrichment increased with disease gene pleiotropy. CLL,.

Because complex diseases tend to show both phenotypic and genotypic overlap [31,32], we hypothesized that the core SuMs would be associated with generally increased susceptibility for all complex diseases. In support of this assertion, we found that the core SuMs from the 13 diseases tended to overlap. This tendency was stronger for core SuMs than SuMs (Figure 3a). The union of the core SuMs was highly enriched for pathways involved in oncological, metabolic and inflammatory complex diseases (Additional file 9). Finally, we tested whether the union of the core SuMs was generally enriched for GWAS genes of various complex diseases. This test comprised 1,570 GWAS genes associated with 145 complex diseases, excluding the genes associated with the 13 complex diseases for which the core SuMs were derived (Additional file 1). We found 2.52-fold enrichment compared to the whole PPI network (*P *< 10^-18^). The enrichment increased if the disease genes were pleiotropic, that is, associated with more than one disease (Figure 3b). For example, when considering only genes associated with more than one disease, we found a 3.1-fold enrichment (*P *< 10^-6^), and when considering genes only associated with more than four diseases we found a 9.1-fold enrichment (*P *< 10^-3^).

## Discussion

We have developed a novel method to define SuMs and core SuMs for complex diseases by combining gene-expression microarray and PPI data. To show the general applicability of the method, we analyzed diseases with highly divergent phenotypes, rather than focusing on a specific subset of phenotypically related diseases. We found that SuMs, and in particular core SuMs, were enriched for GWAS genes. By comparison, no enrichment of GWAS genes was found when analyzing all differentially expressed genes.

To test if core SuMs could be used to find novel genes for functional studies, we analyzed our own gene expression microarray and GWAS data from patients with SAR. This is an optimal model of complex diseases in that it has a clearly defined phenotype that occurs at a given time point each year and the external cause (pollen) and key cell-type (lymphocytes) are known (Extended background in Additional file 2). We identified a SuM and a core SuM, which were reproduced in an independent study. We tested for enrichment of GWAS genes by analyzing a large population-based GWAS. This led to the identification of two novel genes in the core SuM. One of those genes, *FGF2*, was novel in type 1 allergic inflammation. The relevance of this gene was supported by transcriptomal analysis following siRNA-mediated knock-down. The knock-down of *FGF2 *resulted in altered expression of pathways and genes of potential or known relevance for type 1 allergic inflammation. In contrast, we found no enrichment of GWAS genes among all differentially expressed genes. These findings support the hypothesis that core SuMs can be used to identify GWAS genes with moderate effect sizes as well as novel genes for functional studies.

Studies of complex diseases often focus only on specific diseases and the genes associated with them. However, complex diseases show considerable phenotypic and genotypic overlap. Moreover, the effects of individual complex disease genes are generally small, while their collective contribution may be large [33]. This has led to increasing interest in studying groups of diseases and genes [31,32,34,35]. In the final part of our study, we made a corresponding change of scale and considered whether core SuMs from the 13 studied diseases were associated with an increased susceptibility for complex diseases, in general. We found that core SuMs tended to overlap, suggesting that they were involved in shared pathogenic mechanisms in complex diseases. This was supported by the union of the core SuMs being enriched for pathways involved in complex diseases. Finally, we tested if the union of the core SuMs was generally enriched for GWAS genes from 145 complex diseases, which represented hundreds of thousands of patients. Indeed, that union was highly enriched for such GWAS genes. Interestingly, the enrichment was greater when considering only GWAS genes associated with more than one disease. Taken together, our findings showed that the core SuMs of highly interconnected disease genes were associated with increased susceptibility for complex diseases.

We propose that the pathways in the core SuMs increase our understanding of how shared pathogenic mechanisms contribute to complex diseases, and also help explain why many of those diseases show phenotypic overlap [36]. From a therapeutic perspective, the core SuMs may be used to prioritize therapeutic novel genes. It should, however, be noted that even though the number of genes in the core SuMs was considerably smaller than all the differentially expressed genes, it may be difficult to find individual target genes. Instead, perhaps drugs targeting combinations of core SuM genes will be required. Another interesting possibility is that the same novel genes might be exploited to develop drugs that target more than one disease. From a diagnostic perspective, different combinations of SuM and core SuM proteins may be useful as diagnostic markers. We propose that such studies can be performed in both specific disease groups and diseases.

A limitation of this study is that it is mainly based on GWAS genes reported in a public database. This may result in missing GWAS genes in some core SuMs, due to too stringent cutoffs. In this study, we analyzed only intragenic SNPs, while intergenic SNPs may have regulatory and disease-causing roles. Another limitation is that the study is based on known physical and functional PPIs. Thus, our results may be confounded by knowledge bias. Moreover, some of the gene expression microarray studies used in this effort were performed in tissues that contained mixed cell populations. We anticipate that the increasing accuracy of PPIs as well as availability of gene expression microarray and GWAS data will lead to more accurate identification of SuMs.

## Conclusions

SuMs and core SuMs may be used to find novel genes for functional studies, as well as to increase understanding of the specific and shared pathogenic mechanisms in complex diseases and how they relate to phenotypic manifestations.

## Materials and methods

### Definition of susceptibility modules of complex diseases

SuMs were defined by integrating PPI network data and differentially expressed genes for each disease. The PPI network was assembled from a large set of functional and physical PPIs obtained from STRING (version 8, using interactions with a confidence score ≥ 0.7). The SuMs were identified using a step-wise process. First, maximal cliques were extracted from the PPI network (A clique is a complete sub-network, that is, a sub-network with links connecting every pair of its nodes (Figure 1a) [37]. Such a clique is maximal if it is not properly contained within another clique.) For this task we employed our custom clique extraction tools as previously applied [38,39]. We noted that maximal cliques tend to be highly overlapping and we used all cliques down to a minimum size of 2 that were not part of other cliques. Each clique was tested for enrichment (*P *< 0.05) of differentially expressed genes in the disease using Fisher's exact test (Figure 1b). Differentially expressed genes between the patient and control samples were determined using a Student's *t*-test with a *P *< 0.05. Finally, overlapping enriched modules/cliques were mapped onto the PPI network so that each gene was represented only once, and overlapping enriched cliques could be identified as SuMs (Figure 1c). The SuMs included both differentially expressed genes and their neighbors in these cliques. In the SuMs, we identified highly interconnected genes and defined them as core SuMs (Figure 1d). Interconnectivity was measured by calculating the average shortest path length using the Network-Analyzer (v2.6.1) plug-in in Cytoscape (v2.6.0).

### Enrichment of core SuMs for GWAS genes

GWAS genes for all complex diseases were obtained from 'a catalog of published Genome-wide association studies' [40]. We tested for enrichment of GWAS genes using a permutation test. In each permutation, every GWAS gene was replaced with a random gene. The number of random genes present in the corresponding SuMs or core SuMs was noted. This process was repeated 100,000 times. The total number of random genes present in the disease SuMs represented the probability of finding GWAS genes in the SuMs by chance. The significance of the overrepresentation of GWAS-identified genes in the SuMs was measured as the ratio of random permutations that included as many or more SuM genes than the true GWAS-identified genes for all 13 diseases. Similarly, enrichment of GWAS genes in the union of core SuMs was also determined for 145 diseases. Pathway enrichment for the union of core SuM genes was determined using Ingenuity Pathway Analysis [17].

### Gene-expression microarray analysis in seasonal allergic rhinitis

Peripheral blood mononuclear cells from patients with SAR and healthy controls were prepared and stimulated with grass pollen extract or diluent for seven days [20]. For gene expression studies, T helper cells were enriched from the allergen-challenged peripheral blood mononuclear cells using anti-CD4-coated paramagnetic microbeads and a MACS (magnetic cell sorter) system according to the instructions of the manufacturer (Miltenyi Biotec GmbH, Bergisch Gladbach, Germany). cRNA was extracted from 200 ng total RNA using Ambion's Illumina RNA TotalPrep Amplification kit (Ambion, Inc., USA). *In vitro *transcription reaction and cRNA biotinylation were performed overnight (14 h). The RNA/cRNA concentrations where checked using Nanodrop ND-1000 before and after the amplifications. cRNA quality was controlled by BioRad's Experion electrophoresis station (Bio-Rad Laboratories, Inc., CA, USA). Transcriptional profiling in 12 patients was performed using Illumina's Sentrix^® ^Human-6 Expression BeadChips (Illumina Inc., San Diego, CA, USA) according to the manufacturer's instructions. (The data can be obtained from the Gene Expression Omnibus under accession number GSE18574.) Probes with a detection score below 0.95 were discarded prior to differential expression analysis. Differentially expressed genes were determined using *lmFit *from the Bioconductor package *Limma *[41] (Additional file 3). Genes with a *P*-value < 0.05 after correction for multiple comparisons (false discovery rate) were determined to be differentially expressed. To validate results obtained from the data above, an additional set of patients (*n *= 3) was analyzed on an Affymetrix U133A platform as previously described [20].

### GWAS analysis for seasonal allergic rhinitis

This cohort (NFBC1966) included individuals from the provinces of Oulu and Lapland [42]. In 1997 (when participants were aged 31 years), 8,463 survivors were sent postal questionnaires and invited to clinical examination with a 71% response rate. DNA was collected for the majority of participants and a total of 4,772 individuals were successfully genotyped. All aspects of the study were reviewed and approved by the Ethics Committee of the University of Oulu and participants gave written informed consent. Genotyping was done using the Illumina HumanCNV370-Duo chip. The data were imputed to approximately 2.5 million SNPs using NCBI HapMap II CEU build 35 version 21 after pre-filtering SNPs (genotyping rate > 95%, *P*-value for HWE deviation > 10^-4^, minor allele frequency > 1%, imputation with a confidence call of R2 > 0.5) using IMPUTE [43].

The SAR phenotype was defined as a positive response to the 'have you ever had allergic rhinitis' question in the main questionnaire and a positive skin prick test for grass. Controls had a negative response to the question and a negative skin prick test. A positive skin prick test was defined as a mean wheal reaction to grass extract of at least 3 mm. Participants with a positive reaction to negative control (diluent of allergen extracts) or a negative reaction to positive control (10 mg/ml histamine dihydrochloride) were excluded. There were 456 patients that had SAR, while 2,569 individuals were controls.

Association tests for additive effects between SNPs and the defined phenotypes were conducted using the QUICKTEST software and supplemented using R. These analyses were adjusted for sex and relevant principal components of population stratification. Statistically significant SNPs located within and outside the genes of the core SuM were assessed. A SNP was assigned to a gene if that SNP position lies within the start and end region of the gene DNA sequence as defined by the Ensembl database [44]. A higher proportion of SNPs within the genes of the core SuM when compared with the background level of association is indicative of the relevance of the core SuM for the phenotype. The *P*-value of this test is the cumulative probability of finding *k *or more SNPs in that gene subset when the probability of sampling *k *SNPs is given by the hypergeometric distribution. The cutoff of the statistical significance of SNP association was determined to be α = 10^-3 ^via sensitivity analysis of the *P*-values, false discovery rate, true positive rate and odds ratio of the core SuM enrichment (Additional file 6). The false discovery rate was given by *FDR *= *FP/P*, where *P *is the number of positive SNP associations and *FP *is the number of false positive SNP associations within the core SuM under the null hypothesis distribution:

$$FP=\sum_{x}^{m}p\left(x,k,m,n\right)x$$

where *p*(*x*, *k*, *m*, *n*)*x *is the *P*-value of the hypergeometric distribution when *x *out of the *k *significant SNPs are observed within the core SuM, when the core SuM holds *m *of the total *n *SNPs. The number of true positive SNP associations (*TP*) is given by *TP *= *P *- *FP*. The odds ratio of the enrichment was defined as the odds of a significant SNP within the core SuM (or any given subset of genes) as compared to the remaining genes. The linkage disequilibrium within the core SuM is assumed to be similar to the expected linkage disequilibrium in the rest of the genome; therefore, GWAS *P*-value distributions within and outside the core SuM should be comparable.

## Abbreviations

GWAS: genome-wide association studies; PPI: protein-protein interaction; SAR: seasonal allergic rhinitis; siRNA: small interfering RNA; SNP: single-nucleotide polymorphism; SuM: susceptibility module.

## Competing interests

The authors declare that they have no competing interests.

## Authors' contributions

FB and SC were involved in conception, design, acquisition, analysis and interpretation of the data and drafting the manuscript. ALC and LC were involved in conception, design, acquisition, analysis and interpretation of the data. MRJ, RJ and MAL were involved in conception, design and interpretation of the data. AR, GR and HW were involved in acquisition and analysis of the data. MB supervised the entire study and was involved in conception, design, interpretation of the data and drafting the manuscript. All authors were involved in critically revising the manuscript for intellectual content and have given final approval of this version to be published.

## Supplementary Material

Additional file 1 **Additional Table 1 - the genes associated with each disease in the online GWAS catalogue (data obtained on 10 January 2012)**. Click here for file

Additional file 2 **Additional documentation providing Extended background, Extended experimental methods and Extended results**. Click here for file

Additional file 3 **Additional Table 2 - differential expression analysis of allergen-challenged CD4+ cells compared to diluent-challenged controls in SAR**. Click here for file

Additional file 4 **Additional Figure 1 - the SuM associated with seasonal allergic rhinitis**. Click here for file

Additional file 5 **Additional Table 3 - all disease-associated SNPs in the SAR SuM**. Click here for file

Additional file 6 **Additional Figure 2 - sensitivity analysis of the statistical significance level α of a SNP on the Core SuM enrichment**. Click here for file

Additional file 7 **Additional Table 4 - differentially expressed genes in Th2 polarized cells following *FGF2 *knockdown**. Click here for file

Additional file 8 **Additional Figure 3 - analysis of *FGF2 *by siRNA-mediated knock-down of *FGF2 *in Th2 polarized cells, followed by gene expression microarrays**. Click here for file

Additional file 9 **Additional Table 5 - pathways enriched in the combined set of core SuM genes**. Click here for file
