# Supplementary material for: Highly interconnected genes in disease-specific networks are enriched for disease-associated polymorphisms
Source: Genome Biol. 2012 Jun 15;13(6):R46. doi: 10.1186/gb-2012-13-6-r46 (PMC3446318; doi:10.1186/gb-2012-13-6-r46)
Supplement: Additional file 2 — Additional documentation providing Extended background, Extended experimental methods and Extended results. [file gb-2012-13-6-r46-S2.DOC]

Highly interconnected genes in disease-specific networks are enriched for disease-associated polymorphisms

Fredrik Barrenäs, Sreenivas Chavali, Alexessander Couto Alves, Lachlan Coin, Marjo-Riitta Jarvelin, Rebecka Jörnsten, Michael A. Langston, Adaikalavan Ramasamy, Gary Rogers, Hui Wang, Mikael Benson

**ADDITIONAL DOCUMENTATION**

****EXTENDED BACKGROUND****

Seasonal allergic rhinitis as a disease model

As a brief background, the analysis of complex diseases is complicated by variable disease phenotypes and disease course. Moreover, the external causes and the key cells may be unknown or difficult to obtain from patients. Seasonal allergic rhinitis (SAR) may be an optimal model for complex diseases because the phenotype is well defined and occurs at a given time point each year. The external cause (pollen) and key cell (type 2 T helper (Th2) cells) and a read-out (Th2 cytokines) are known . In allergic patients, allergen-challenge results in Th2 cell activation, which result in the release of Th2 cytokines that activate different inflammatory effector cells. The activation of Th2 cells can be mimicked *in vitro,* by challenging peripheral blood mononuclear cells (PBMC) with the relevant allergen. In this way T cells are activated by antigen presenting cells, as is the case *in vivo.* The efficacy of the allergen-challenge can be tested by measuring the protein levels of Th2 cytokines in the cell supernatants. CD4 + T cells are then purified and analyzed with gene expression microarrays. However, in this model the complex interactions between antigen presenting cells and T cells makes functional studies impractical. Therefore, functional studies with siRNA followed by gene expresson microarrays were performed in Th cells that had been Th2 polarized as described above.

EXTENDED experimental methods

*FGF2* knockdown

CD4+ T cells were purified from buffy coat from six healthy subjects using CD4+ T cell isolation kit II (Miltenyi, Bergisch-Gladbach, Germany). Freshly isolated CD4+ T cells were electroporated with 500 mM scrambled control siRNAs (Dharmacon, Lafayette, CO, USA ) or 500mM ON-TARGETplus SMARTpool siRNAs targeting FGF2 (Dharmacon, Lafayette, CO, USA ) by a nucleofectorTM II Device (Lonza Ltd., Basel, Switzerland). After 5 hours, cells were polarized towards Th2 direction by plate-bound anti-CD3 (500 ng/mL), soluble anti-CD28 (500 ng/mL), IL-4 (10 ng/mL) and anti-IL-12 (5 ug/mL) in the presence of IL-2 (17 ng/mL). After another 48 hours, cells were harvested and the gene expression analysis was performed using the SurePrint G3 Human GE 8x60k Microarray kit (Aglient Technologies, Palo Alto, CA, USA) according to the manufacturer’s instructions. Differentially expressed genes resulting from the knockdown were determined using the Bioconductor package LIMMA. The result from the differential expression analysis can be found in additional table 4.

Network- and pathway analysis of gene expression microarray data

The Ingenuity Pathway Analysis tool (IPA) was used to organize differentially expressed genes into networks of interacting genes and to find modules of functionally related genes that correspond to pathways. The IPA consists of a global network that is based on reviewing and extracting mammalian gene interactions from review of more than 200,000 articles in 275 peer reviewed journals. The findings for the IPA were manually curated and assigned to ontologic classes by content and modeling experts. Interactions were also inferred algorithmically by combining functional and structural information about proteins. Every interaction can be referenced back to a MedLine ID within the application. Identification of networks formed by the differentially expressed genes was done in a stepwise manner:

1. Differentially expressed genes are scored on the basis of the number of specific interactions found within the IPA global network. Focus genes were identified as those having direct or indirect interactions with other genes in the IPA database.

2. The specificity of connections for each focus gene was calculated by the percentage of its connections to other differentially expressed genes. A network was then constructed, starting with the gene with the highest specificity of connections. Each network contained a maximum of 35 genes. This process was repeated for the remaining genes to construct new networks. As the number of focus genes is reduced as a result of their inclusion in previous networks, genes from the global network not in the original list but with significant specific connectivity to the growing network can be included into those new networks.

3. A statistical algorithm based on the Fischer exact test was used to rank the networks. Identification of pathways was based on using canonical pathways in the IPA as templates for the differentially expressed genes. Again using the Fischer exact test, the resulting pathways were ranked according to their correlation with the template in the IPA.

EXTENDED RESULTS

Enrichment of disease-associated SNPs in SAR core SuM

To make the analysis robust we applied negative and positive controls. Negative controls were applied to rule out the hypothesis that the any predefined set of genes had an overrepresentation of significant SNPs compared to the background. To this effect, we tested the genes in the interactome, differentially expressed genes in allergen-challenged CD4+ cells, and the SuM genes against the background (whole chip). Lesser significance, if any, of genes in negative controls compared to the core SuM genes is expected. Positive controls are applied to confirm that SNPs within genes of the core SuM are overrepresented if compared with SNPs within the gene set of the interactome, the differentially expressed genes in allergen-challenged CD4+ cells and all the SuM genes. These tests assess the probability that this or a more extreme number of SNPs found in a given subset of genes (e.g. core SuM, PPI network, differentially expressed genes, SuM) occurred by chance. Further, the genes in the core SuM were significantly enriched for disease-associated polymorphisms compared to all the positive controls used (differentially expressed genes and SuM genes).

| **Experiment** | **Subset** | **P value** | **OR** |
| --- | --- | --- | --- |
| Main hypothesis | Core SuM | 1.01E-05 | 3.45 |
| Negative controls | PPI network | 0.005 | 1.12 |
|  | Differentially expressed genes | 0.986 | 0.84 |
|  | SuM | 0.996 | 0.65 |
| Positive controls | Core SuM/PPI network | 2.80E-05 | 3.21 |
|  | Core SuM/Differentially expressed genes | 6.28E-07 | 4.45 |
|  | Core SuM/SuM | 4.90E-10 | 10.29 |

False Discovery Rate calculation

To assess how many how many real disease genes can be identified for a given number of genes in coreSuM, we carried out a false discovery rate calculation.  We calculated the expected number of genes expected by chance alone to be enriched in the module, using the hypergeometric distribution.  We find that the median number of genes enriched under the hypergeometric distribution is 0.0 and the expected number (or mean) is 0.46.  Hence the expected FDR for the two genes selected is 23%.  We have carried out a second FDR analysis on the SNPs themselves, using the procedure of Storey & Tibshirnai [] to calculate FDR for all SNPs contained in the core module.  In this way we found 4 genes, all inside the FGF2 gene with FDR < 5%

| SNP | FDR (Q values) |
| --- | --- |
| rs7667439 | 0.0284 |
| rs11098659 | 0.0027 |
| rs7673567 | 0.0284 |
| rs11725823 | 0.0027 |

This evidence strongly supports strongly the association of the FGF2 gene as a new allergy susceptibility gene.

Analysis of gene expression microarray data from CD4 + cells from patients with SAR and healthy controls

To obtain a functional overview of the large number of genes that were differentially expressed by allergen in patients and controls we performed pathway analysis, using the IPA software. We first analysed allergen challenged CD4 + cells from patients with SAR, compared to diluent-challenged controls. The allergen challenge response was seen as a model of type 1 allergic inflammation. Pathway analysis of the gene expression microarray data showed that the most significant pathways of potential relevance for type 1 allergic inflammation were *glucocorticoid receptor signaling* (*P* < 0.01), *ERK/MAPK signaling* (p<0.01), *Death receptor signaling* (p<0.01), *IL4 signaling* (p<0.01), *PKC signaling in T lymphocytes* (p<0.01) and *NFAT signaling* (p<0.01) These pathways included known disease genes in type 1 allergic inflammation, such as *CD28, CD86, GZMB, NFATC1, NFATC2* and *STAT6.* Pathway analysis of gene expression microarray data from allergen challenged CD4 + cells from patients compared to healthy controls, showed that the most significant pathways of relevance for type 1 allergy were *leukocyte extravasation* (*P* < 0.05), *T cell signaling* (p<0.05), *CD40 signaling* (p<0.05), *JAK-STAT signaling* (p<0.05) *and FGF signaling* (*P* < 0.05). These pathways included known disease genes in type 1 allergic inflammation, such as IL16 , IL17RB and IL21 .

Analysis of *FGF2* by siRNA-mediated knock-down of *FGF2* in Th2 polarized cells, followed by gene expression microarrays

A background about seasonal allergic rhinitis and Th2 cells is given in a separate section, below. Briefly, the rationale behind these experiments was to examine the relevance of *FGF2* for allergy, by testing if siRNA-mediated knock-down resulted in altered expression of pathways and genes of potential or known relevance for allergy. We found that siRNA-mediated knockdown of *FGF2* in Th2-polarized cells resulted in significantly decreased expression of *FGF2*. To identify FGF2-induced pathways of relevance for allergy, we performed gene expression microarray analysis following knockdown. 146 genes were significantly differentially expressed (Additional table 4).

Pathway analysis of the gene expression microarray data showed that the most significant pathways of potential relevance for allergy were *TREM-1 signalling* (*P* < 0.01), *IL-17A-signaling in airway cells* (*P* < 0.01) and *IL-17F signalling in allergic inflammatory airway disease* (*P* < 0.05) (Additional figure 3).

Additional REFERENCES

1. Benson M, Adner M, Cardell LO: **Cytokines and cytokine receptors in allergic rhinitis: how do they relate to the Th2 hypothesis in allergy?** *Clin Exp Allergy* 2001, **31**(3):361-367.

2. Storey JD, Tibshirani R: **Statistical significance for genomewide studies**. *Proc Natl Acad Sci U S A* 2003, **100**(16):9440-9445.

3. Bandeira-Melo C, Sugiyama K, Woods LJ, Phoofolo M, Center DM, Cruikshank WW, Weller PF: **IL-16 promotes leukotriene C(4) and IL-4 release from human eosinophils via CD4- and autocrine CCR3-chemokine-mediated signaling**. *J Immunol* 2002, **168**(9):4756-4763.

4. Wang H, Mobini R, Fang Y, Barrenas F, Zhang H, Xiang Z, Benson M: **Allergen challenge of peripheral blood mononuclear cells from patients with seasonal allergic rhinitis increases IL-17RB, which regulates basophil apoptosis and degranulation**. *Clin Exp Allergy* 2010, **40**(8):1194-1202.

5. Suto A, Nakajima H, Hirose K, Suzuki K, Kagami S, Seto Y, Hoshimoto A, Saito Y, Foster DC, Iwamoto I: **Interleukin 21 prevents antigen-induced IgE production by inhibiting germ line C(epsilon) transcription of IL-4-stimulated B cells**. *Blood* 2002, **100**(13):4565-4573.

6. Corrigan CJ, Wang W, Meng Q, Fang C, Eid G, Caballero MR, Lv Z, An Y, Wang YH, Liu YJ *et al*: **Allergen-induced expression of IL-25 and IL-25 receptor in atopic asthmatic airways and late-phase cutaneous responses**. *J Allergy Clin Immunol* 2011, **128**(1):116-124.

7. Wu M, Peng A, Sun M, Deng Q, Hazlett LD, Yuan J, Liu X, Gao Q, Feng L, He J *et al*: **TREM-1 amplifies corneal inflammation after Pseudomonas aeruginosa infection by modulating Toll-like receptor signaling and Th1/Th2-type immune responses**. *Infect Immun* 2011, **79**(7):2709-2716.

8. Lajoie S, Lewkowich IP, Suzuki Y, Clark JR, Sproles AA, Dienger K, Budelsky AL, Wills-Karp M: **Complement-mediated regulation of the IL-17A axis is a central genetic determinant of the severity of experimental allergic asthma**. *Nat Immunol* 2010, **11**(10):928-935.
