# Supplementary material for: Highly interconnected genes in disease-specific networks are enriched for disease-associated polymorphisms
Source: Genome Biol. 2012 Jun 15;13(6):R46. doi: 10.1186/gb-2012-13-6-r46 (PMC3446318; doi:10.1186/gb-2012-13-6-r46)
Supplement: Additional file 4 — Additional Figure 1 - the SuM associated with seasonal allergic rhinitis. [file gb-2012-13-6-r46-S4.PDF]

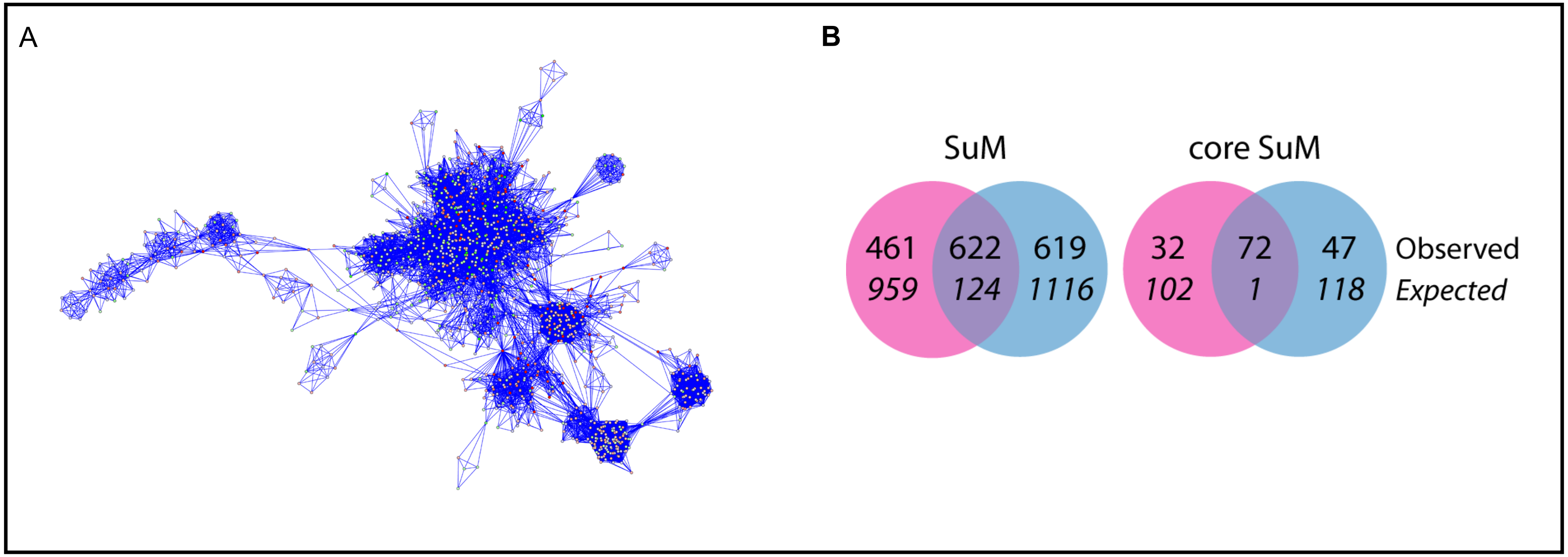

Additional Figure 1: The SuM associated with seasonal allergic rhinitis.

A) SAR SuM clearly showing the underlying cliques. The core SuM is located in the largest cluster in the middle of the SuM. 2822 differentially expressed genes were represented in the PPI network; the SAR SuM included 622 differentially expressed genes. In total, the SAR SuM consisted of 1191 genes and 18761 PPIs (Supplemental figure 1). The genes with the highest number of interactions were TNF (n=178), followed by IFNG (n=167), BCL2 (n=148) and IL1B (n=144).

B) SAR SuM and core SuM are reproducible. To test the reproducibility of the SAR SuM and core SuM, another SuM was constructed from another dataset of CD4+ cells that had undergone the same allergen challenge. The two sets were obtained at different time points and were analyzed with different microarray platforms. The pink circle in the Venn diagram represents the first set which was used throughout this study and the blue the second set, which was used for validation. The repeated analyses resulted in highly similar SuMs and core SuMs ($p < 10-15$ in both cases, determined by $\chi 2$-test).
