# Supplementary material for: Highly interconnected genes in disease-specific networks are enriched for disease-associated polymorphisms
Source: Genome Biol. 2012 Jun 15;13(6):R46. doi: 10.1186/gb-2012-13-6-r46 (PMC3446318; doi:10.1186/gb-2012-13-6-r46)
Supplement: Additional file 6 — Additional Figure 2 - sensitivity analysis of the statistical significance level α of a SNP on the Core SuM enrichment. [file gb-2012-13-6-r46-S6.PDF]

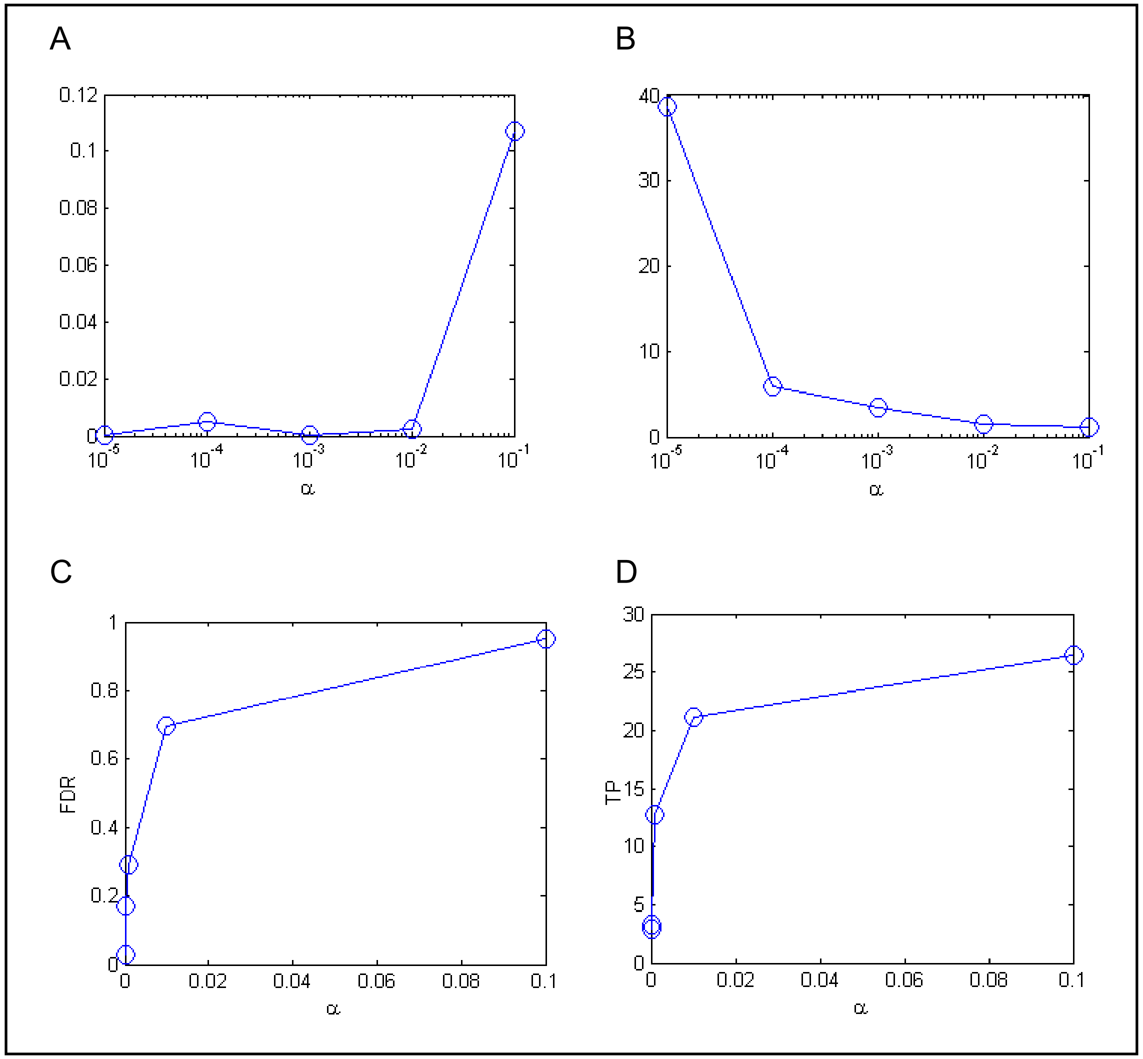

Additional Figure 2: Sensitivity analysis of the statistical significance level α of a SNP on the Core SuM enrichment.

(A) P-value of the enrichment. (B) Odds ratio. (C) False discovery rate. (D) Expected number of true positive SNP associations within the module.

For the case study of seasonal allergic rhinitis, sensitivity analysis was employed to assess the robustness of the enrichment of disease-associated SNPs in the core SuM. The sensitivity analysis of the GWAS significance level α showed that for α<0.01 the enrichment p-values were consistently small and nominally significant (p<2.4×10-3). Furthermore, the odds-ratio significantly increased with decreasing of α as well as FDR and the number of expected true positives (TP). The core SuM FDR reached nominal statistical significance (FDR=0.03, TP≈3) for a cut-off α=10-5. The cut-off of the statistical significance level of the GWAS was set to α=10-3 since it was a balanced compromise between the number of TP, FDR, p-value and OR.
