# Supplementary material for: Highly interconnected genes in disease-specific networks are enriched for disease-associated polymorphisms
Source: Genome Biol. 2012 Jun 15;13(6):R46. doi: 10.1186/gb-2012-13-6-r46 (PMC3446318; doi:10.1186/gb-2012-13-6-r46)
Supplement: Additional file 8 — Additional Figure 3 - analysis of FGF2 by siRNA-mediated knock-down of FGF2 in Th2 polarized cells, followed by gene expression microarrays. [file gb-2012-13-6-r46-S8.PDF]

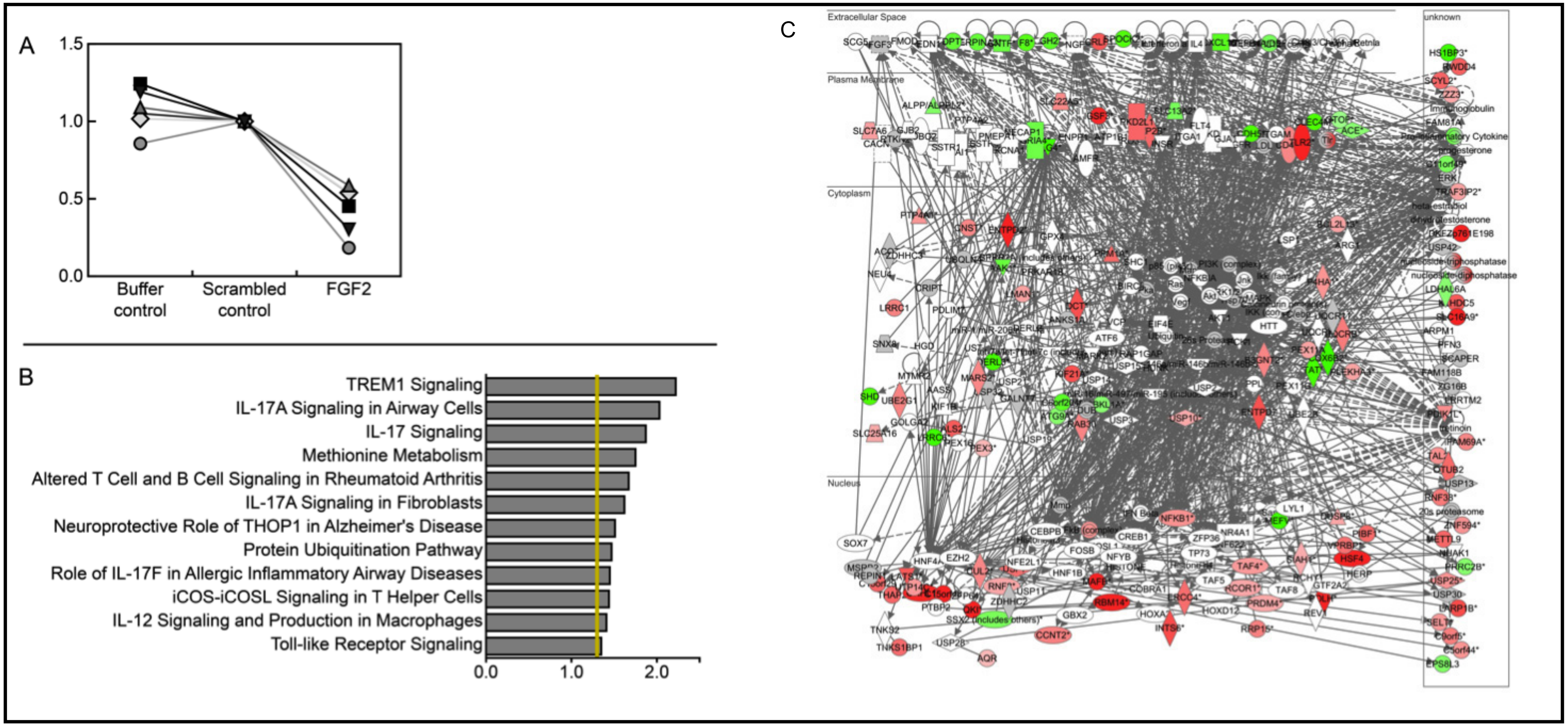

Additional Figure 3: Analysis of FGF2 by siRNA-mediated knock-down of FGF2 in Th2 polarized cells, followed by gene expression microarrays

A) FGF2 expression was knocked down by 57% in Th2 polarized cells from 6 donors ($P < 0.001$).

B) Pathway analysis of the genes affected by FGF2 knockdown showed that the most significant pathways of relevance for allergy were TREM-1 signaling ($P < 0.01$), IL-17A-signaling in airway cells ($P < 0.01$) and IL-17F signaling in allergic inflammatory airway disease ($P < 0.05$).

C) Network representation of differentially expressed genes in Th2 cells following siRNA mediated knock-down of FGF2. The interaction network was generated using Ingenuity pathway analysis. Genes shaded in red were up-regulated whereas green were down-regulated. The color intensity reflects the significance value of the regulation. Solid and dotted lines represent direct and indirect interactions, respectively. Node shapes represent the class of molecule, for example donut shapes represent transcription factors (for detailed information see, http://www.ingenuity.com)
